# Health Problems while Working as a Volunteer or Humanitarian Aid Worker in Post-Earthquake Nepal

**Published:** 2018-06-30

**Authors:** Durga Bhandari, Prativa Pandey

**Affiliations:** 1 CIWEC Hospital and Travel Medicine Center, Kapurdhara Marg, Kathmandu, Nepal; 2 Department of Internal Medicine, Kantipur Dental College Teaching Hospital and Research Center, Kathmandu, Nepal

**Keywords:** *aid worker*, *diarrhea*, *earthquake*, *Nepal*, *traveler*, *volunteer*

## Abstract

**Introduction:**

Volunteers and humanitarian aid workers working in disaster struck areas of the world are a vulnerable group of travelers. Nepal saw an influx of these humanitarian aid workers following earthquakes in April and May 2015. This study was undertaken to find out the pre-travel preparation and to estimate the risk of disease while the volunteers were deployed in Nepal.

**Methods:**

This was a descriptive cross-sectional study conducted at CIWEC Hospital located in Kathmandu. A questionnaire was given to all volunteers and aid workers who arrived at the hospital for evaluation of health related problems and agreed to be part of the study.

**Results:**

Ninety-five volunteers were enrolled in the study. Among these, 65 (68%) were female and 30 (32%) were male. The immunizations received before travel were Hepatitis A 82 (86%), Hepatitis B 82 (86%), Typhoid 70 (73%), Rabies 38 (40%), Japanese Encephalitis 34 (36%), Influenza within last one year 23 (24%), measles 48 (51%), Cholera 34 (36%), Tetanus within 10 years 71 (75%) and Varicella 38 (40%). Forty-four (45%) of travelers carried medication for treatment of Traveler's Diarrhea (TD) which included Ciprofloxacin, Azithromycin, Loperamide and others like Metronidazole and Charcoal. The common illnesses encountered were gastrointestinal, skin problems, injury and musculoskeletal problems, respiratory problems, genitourinary problems, cardiovascular, psychological problems, syncope, and miscellaneous.

**Conclusions:**

Traveler's Diarrhea and dermatological problems were the most common health related problems. Volunteers were not properly prepared for self-treatment and pre-travel preparation was sub-optimal. Important pre travel health advice will decrease the incidence of health problems in this group.

## INTRODUCTION

An earthquake of magnitude 7.8 Richter scale struck Nepal at noon on 25th April 2015 killing 9000 people, injuring more than 22000, displacing two million persons and destroying about 1000 health facilities.^13^ A second major aftershock occurred two weeks later with a magnitude of 6.8.^1^ Volunteers and aid workers from different countries arrived in Nepal to complement the relief work done by the Government, the Nepali people and various international governmental organizations. Travelling through difficult terrain and harsh conditions, volunteers were exposed to various health hazards. Planning and training are critical to optimal emergency response.^4^ We conducted this study to find out the perceived risks and preparedness prior to working as a volunteer in earthquake affected areas in Nepal and to evaluate disease conditions that affected these volunteers while working as a relief worker. These volunteers were mostly working in villages away from Kathmandu in a non- medical setting.

## METHODS

The study was a descriptive cross-sectional study conducted in CIWEC Hospital, Kathmandu, Nepal from May to November 2015. The study group included volunteers and aid workers involved in post-earthquake rehabilitation work, who presented to the clinic with health related problems and agreed to be part of the study. They completed a questionnaire that included age, sex, nationality, profession at home or skills brought to the job, duration of stay, organization involved, health consultation prior to arrival, immunization, perceived health risks, safe water practices, mosquito bite prevention, chemoprophylaxis, any pre-existing health problems and current health problems. The information was verified by a physician and confirmed during the period of clinic visit. Data was collected and managed in Microsoft Excel and descriptive analysis was done.

## RESULTS

Ninety-five patients were included in the study; 65 (68%) were female and 30 (32%) were male. The average age of the study group was 30 yrs. with a range of 16–65yrs. The mean duration of stay in Nepal was 73 days (4–365days). Most of the volunteers were from United States, United Kingdom, Germany, Australia, Switzerland, Denmark and Spain. About 29 (31%) of the volunteers were skilled workers; they were engineers, architects, electricians concerned with building shelters/school/health posts and some were mental health counselors whereas 66 (69%) were non-skilled. Among these volunteers, 77 (81%) were part of an organization and 18 (19%) of the volunteers had arrived on their own.

**Figure 1.**
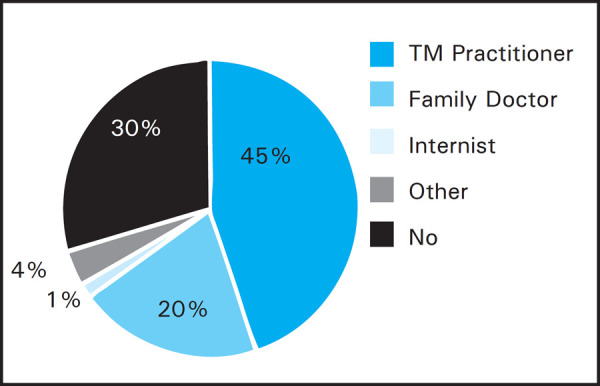
Pre-travel health assessment.

Sixty-seven patients (71%) had received a health consultation prior to departure. Among them 30 (45%) had visited a travel medicine (TM) practitioner; 20 (30%) had seen a family doctor, Internist 1 (1%), 3 (4%) received information from the internet, travel agents or their own organization and 13 (20%) of the volunteers did not receive any health advice prior to arrival (Figure 1).

Eighty-two (86 %) of the study participants were vaccinated with Hepatitis A and Hepatitis B. Seventy (73%) had typhoid vaccination. Pre-Exposure immunization for rabies was 38 (40%). Other vaccine rates were 34 (36%) for JE (Japanese encephalitis), 23 (24%) for flu, 48 (51%) for measles, 34 (36%) for cholera, 71 (75%) for tetanus and 38 (40%) for varicella (Table 1).

**Table 1.** Percentage of traveller immunized against different vaccines.

Vaccines (n = 95)	YES n (%)	NO n (%)
Hepatitis A	82 (86)	13 (14)
Hepatitis B	82 (86)	13 (14)
Typhoid	70 (73)	25 (27)
Rabies	38 (40)	57 (60)
JE	34 (36)	61 (66)
Flu	23 (24)	72 (76)
Measles	48 (51)	47 (49)
Cholera	34 (36)	61 (64)
Tetanus	71 (75)	24 (25)
Varicella	38 (40)	57 (60)

**Figure 2.**
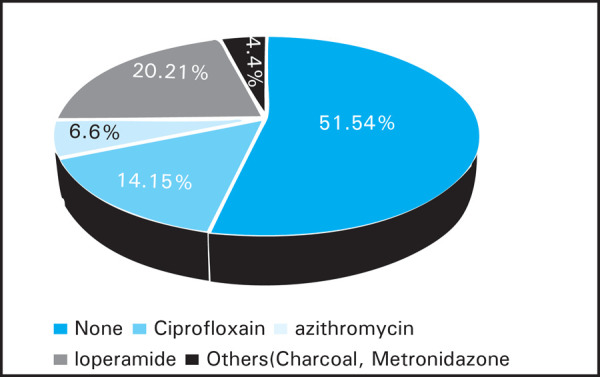
Percentage of travellers carrying medications for traveller's diarrhea.

About half of the volunteers carried medications for treatment of traveler's diarrhea which included ciprofloxacin, Azithromycin, Loperamide and others like metronidazole and charcoal (Figure 2).

Total 84% of the volunteers used protection against insect bites, 63% of volunteers used insect repellent creams and 31% used bed nets. About 2 (2%) were taking malaria chemoprophylaxis with malarone or doxycycline. Others 4 (4%) were using repellents like mint cream paste, citronella herb and claimed natural product (enoxolone cream).

**Figure 3.**
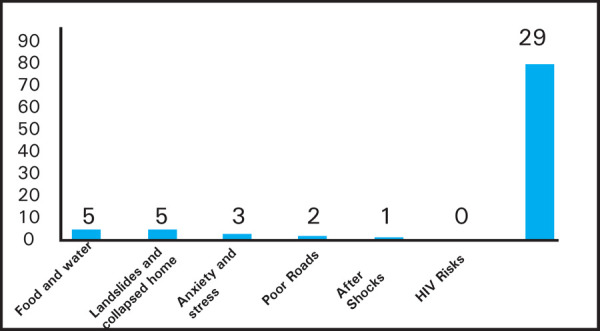
Perceived risk among volunteers.

Seventy-nine volunteers said they did not perceive volunteering in earthquake areas as a risk. About 5 (6%) said they were concerned about food and water hygiene, landslides and collapsed homes; 3 (3%) for anxiety and stress; 2 (2%) for poor roads, 1 (1%) for after shocks. There was no perceived risk of sexually trasmitted disease such as HIV (Figure 3).

**Figure 4.**
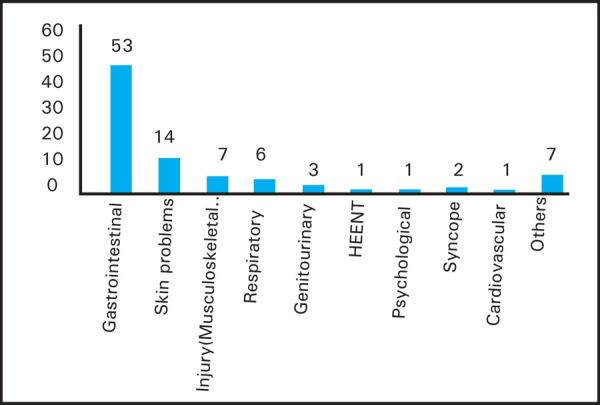
Disease among study population.

Among ninety-five volunteeres who came to CIWEC Hospital for medical advice, the complaints included: 53 (56%) had gastrointestinal illness; 14 (15%) had skin illness, 7 (8%) had injury/musculoskeletal illness, 6 (6%) had respiratory problems, 3 (3%) had genitourinary illnes 2 (2%) had syncope during work and 1 (1%) had eye infection, psychological symptoms and cardiovascular illness and others 7 (7%). Others included single case of anaphylaxis etiology unknown, foreign body in ear and health advice (Figure 4).

## DISCUSSION

The earthquakes of April 25 and May 12, 2015 in Nepal resulting in almost 9000 deaths, 23000 injuries, over 2 million displaced persons and destruction of 1000 health facilities were followed by multiple aftershocks which hindered the rescue efforts. ^2^ Many houses were flattened in districts like Sindhupalchok, Dhading, Gorkha, Rasuwa, Nuwakot and Bhaktapur. Massive avalanches were triggered in the Everest area and in Langtang resulting in hundreds of dead and injured, both Nepali's and foreign travelers. Help started to pour in immediately from neighboring countries and from non governmental organizations. Many other countries also sent in relief crew and material aid. Outside help is needed in the case of a large scale emergency such as the Nepal earthquake because the country's resources are overwhelmed and damaged health facilities become dysfunctional.^5^ While the immediate need post-earthquake seems to be for rescue teams, food and shelter and medical personnel including trauma surgeons, orthopedic surgeons, anesthesiologists etc, community rebuilding goes on for months to years and strong community involvement is key to success in most disaster situations.^3,4^

Volunteers provide much needed extra man power and surge capacity to deal effectively in a disaster situation if properly utilized although volunteerism has its own benefits and challenges.^6,7^ There are many challenges to volunteering overseas^8^ but in spite of that volunteerism particularly “digital volunteerism” for enhanced communication is on the rise.^7^ Preparing foreign volunteers to work in a community in a disaster area is dependent upon perceived risks in that area for the volunteers. A large portion of the respondents (79%) said they did not perceive any risk while working in earthquake affected areas in Nepal while 5% worried about food and water hygiene and an equal number worried about landslides and collapsed homes. 31% of the volunteers were skilled workers in that they were engineers, architects, electricians, mental health counselors and the rest were mainly students or administrative support staff. Self- efficacy or one's belief in one's ability to perform a specific task was found to be the primary predictor of willingness to respond regardless of severity of disaster in a cohort of medical reserve corps volunteers.^9^

Nepal is a country with high risk of gastrointestinal infections like enterically transmitted hepatitis and typhoid.^10–13^ The risk of typhoid fever and paratyphoid fever among travelers to Nepal is among the highest in the world, and the prevalence of fluoroquinolone resistance is high.^14^ Typhoid vaccine is recommended for all travelers to Nepal. Most of the volunteers did receive immunizations for hepatitis A 82 (86%) and typhoid 70 (76%) whereas the vaccine uptake for Rabies was 38 (40%) and for JE 34 (36%). Animal bites are common in Nepal with risk of rabies exposure. ^15–17^ Pre-exposure rabies vaccine is desirable but not a must since effective post-exposure prophylaxis is available in the country. Cholera vaccine uptake was 34 (36%). Although Nepal is endemic for Cholera with outbreaks from time to time, there have been no reports of cholera in foreign travelers and cholera vaccine is not routinely advocated. ^18–20^ JE is endemic in Nepal, with highest disease risk occurring in the Terai region during and immediately after the monsoon season (June through October). JE has been identified in local residents of the Kathmandu Valley,^21–22^ and there has been a single case reported in a foreigner.^23^ JE vaccine is recommended for extended stay in Kathmandu particularly with rural exposure as was the case with our volunteers. Volunteers had low uptake of vaccine preventable diseases like Measles and Varicella. Traveler's diarrhea (TD) is the most common problem among travelers to Nepal for years.^20–25^ Diarrhea was still the most common morbidity among Israeli Defense Force response team during post earthquake mission in Kathmandu.^26^

About 70% of volunteers did see a medical practitioner before the trip. More than half (55%) were not carrying any medications for self treatment of TD. Only 6.6% were given azithromycin for stand by TD treatment compared to 14% who were given ciprofloxacin. Campylobacter is the number one pathogen causing TD in Nepal and it is highly resistant to ciprofloxacin.^27–28^ Malaria is not a risk for most travelers to Nepal.^29^ Two of our volunteers were taking malaria chemoprophylaxis and most were using mosquito bite protection which is all that should be required.

The limitatations of the study was that it was a hospital based study which captured only those presenting for other illnesses- so we couldn't include all the aid workers who arrived in Nepal. A larger study among returned aid workers would be very useful. It would also be useful to compare these results to a matched group to see whether there are unique problems in the volunteer group. The sample size was not decided prior to the study as we tried to enroll as many patients as we could.

## CONCLUSIONS

It was satisfying to see that there was greater than 75% coverage in immunization against hepatitis AB and typhoid, and a good level of understanding of food and water safety in Nepal, however a surprising number of this cohort did not perceive that disaster relief work could pose a risk to health. Additionally, gastrointestinal illness remained the most common medical problem in this group, which is consistent with other traveler cohorts to Nepal.^12–14^ Only a small number carried azithromycin for empirical self-treatment of TD, which is the gold standard of treatment in this region.
